# Biocompatibility enhancement via post-processing of microporous scaffolds made by optical 3D printer

**DOI:** 10.3389/fbioe.2023.1167753

**Published:** 2023-04-12

**Authors:** Jurga Jeršovaitė, Ugnė Šarachovaitė, Ieva Matulaitienė, Gediminas Niaura, Daiva Baltriukienė, Mangirdas Malinauskas

**Affiliations:** ^1^ Laser Research Center, Faculty of Physics, Vilnius University, Vilnius, Lithuania; ^2^ Institute of Biochemistry, Life Sciences Center, Vilnius University, Vilnius, Lithuania; ^3^ Department of Organic Chemistry, Center for Physical Sciences and Technology, Vilnius, Lithuania

**Keywords:** 3D printing, photopolymerization, stem cells, tissue engineering, post-processing, biocompatibility, differentiation

## Abstract

Providing a 3D environment that mimics the native extracellular matrix is becoming increasingly important for various applications such as cell function studies, regenerative medicine, and drug discovery. Among the most critical parameters to consider are the scaffold’s complicated micro-scale geometry and material properties. Therefore, stereolithography based on photopolymerization is an emerging technique because of its ability to selectively form volumetric structures from liquid resin through localized polymerization reactions. However, one of the most important parameters of the scaffold is biocompatibility, which depends not only on the material but also on the exposure conditions and post-processing, which is currently underestimated. To investigate this systematically, microporous scaffolds with pore sizes of 0.05 mm^3^ corresponding to a porosity of 16,4% were fabricated using the stereolithography printer *Asiga PICO2 39 UV* from the widely used resins *FormLabs Clear* and *Flexible*. The use of various polymers is usually limited for cells because, after wet chemical development, the non-negligible amount of remaining monomers intertwined in the photopolymerized structures is significantly toxic to cells. Therefore, the aim of this research was to find the best method to remove monomers from the 3D scaffold by additional UV exposure. For this purpose, a Soxhlet extractor was used for the first time, and the monomers were immersed in different alcohols. A Raman microspectroscopy was also used to investigate whether different post-processing methods affect DC (cross-linking) to find out if this specifically affects the biocompatibility of the scaffolds. Finally, mesenchymal stem cells from rat dental pulp were examined to confirm the increased biocompatibility of the scaffolds and their ability to support cell differentiation into bone tissue cells.

## 1 Introduction

Cell cultures are a powerful *in vitro* tool for studying cell function, tissue morphology and disease mechanisms, drug action, protein production, and tissue engineering development. The commonly used 2D cultures have many limitations, such as disruption of interactions between the cellular and extracellular environments and changes in cell morphology, polarity, protein and gene expression (Caleb and Teng, 2020). Various three-dimensional (3D) cell culture techniques are being developed to overcome these drawbacks and are expected to bridge the gap between 2D cell cultures and animal models (Kapałczyńska et al., 2018; Baruffaldi Désirée et al., 2021).

The assembly of multilayered 3D cell structures can be achieved using printed scaffolds. They are microorganized supports that strongly influence the properties and behaviour of the cells (Knight Eleanor, 2015). A suitable scaffold must mimic the microenvironment *in vivo* and support cell adhesion, proliferation, migration, and differentiation.

Among the techniques proposed for the fabrication of scaffolds, precise control of the scaffold structure can be provided by photopolymerization methods such as Laser Driect Writing (LDW) (Danilevicius et al., 2012; Weisgrab et al., 2020; Sharaf et al., 2022), Stereolithography/Direct Light Processing (SLA/DLP) (González et al., 2020; Hart et al., 2020; Ao-Ieong et al., 2021; Bayarsaikhan et al., 2021; 2022; Hwangbo et al., 2021), holographic lithography (Stankevicius et al., 2012) and others. SLA and DLP are the two most widely used technologies in commercial UV desktop printers. Due to their high resolution, precision, accuracy and speed, it allows the creation of complex 3D structures ranging from micrometer -sized needles to life-sized organs (Lakkala et al., 2023). Three-dimensional (3D) printing, also known as additive manufacturing (AM), builds objects layer by layer, replicating the computer-aided design (CAD) model and forming geometries by selectively solidifying a liquid resin through photopolymerization reactions. UV light triggers radical polymerization - the cross-linking of monomer-to-polymer chains - which offers many advantages such as low energy and temperature requirements and short curing times (Tehfe et al., 2013). Therefore, 3D printing has not only attracted great interest in regenerative medicine (Malinauskas et al., 2012; Bertana et al., 2020) and tissue engineering but can also be used in various fields such as electronics, mass-customized production, dentistry, prototyping and other (Bikas et al., 2016), as it can produce complex geometries by adjusting the appropriate pre-polymer material compositions and processing conditions.

3D scaffolds can be made from various types of natural and synthetic materials. Natural materials are advantageous for various applications, including tissue engineering, because they control cellular functions properly and are biodegradable. Synthetic materials have defined chemical structures and tunable mechanical properties. In addition, scaffolds made from synthetic materials offer high reproducibility. Although not all synthetic materials are biodegradable, both biodegradable and non-biodegradable materials can be used for biomedical applications. However, poor biocompatibility, functionality, and invalid physicochemical as well as biomechanical properties may limit their use for such applications. The vast majority of synthetic photocurable materials (photoresins) used for 3D printing typically contain (meth)acrylates, photoinitiators, and additives that are usually toxic to cells (Zhu et al., 2015; Oskui et al., 2016). During polymerization, the double carbon bonds (C=C) of photoresin monomers convert to a single carbon bond (C-C) to form a polymer (Pianelli et al., 1999; Baldacchini et al., 2020).

Nevertheless, complete polymerization often does not occur. Incomplete polymerization is a significant problem because unpolymerized monomers remain on the scaffold, making them non-biocompatible with cells and tissues. For this reason, many post-processing methods are used to wash out and/or crosslink the residual monomers to complete the polymerization process after printing, which can play a crucial role in the outcome. Post-washing protocols used in many studies include simple rinsing in solvents (Tayalia et al., 2008; Grigaleviciute et al., 2020; Xu et al., 2021; Potrich et al., 2022), the use of rotary washing machines, and the use of ultrasonic baths (Hart et al., 2020; Jin et al., 2022), which lead to different biocompatibility results depending on the washing time and temperature. Previous studies have shown that ultrasonic baths have a better washing result (Jin et al., 2022), in the elution of monomers and that longer times usually increase cell viability values (Grigaleviciute et al., 2020; Xu et al., 2021; Jin et al., 2022). One of the factors limiting the leaching of residual monomers from printed samples is soluble impurities after some time of washing. For this reason, in this study, we introduce washing of the monomer with a Soxhlet extractor - a continuous renewal of the fresh solvent.

UV post-curing is also effective in improving the biocompatibility of polymers (Hart et al., 2020), and similar to post-washing methods. It increases over the time, but only up to a certain point (Bayarsaikhan et al., 2022). In addition, previous studies have shown that different post-curing devices with different wavelengths and temperatures also affect the biocompatibility of the specimens (Kim et al., 2022). It is important to note that the above studies have also shown that the degree of polymerization, which is calculated as the degree of conversion (DC), also depends on the UV post-curing parameters. Moreover, UV light can be used as an additional treatment procedure with two tasks - to crosslink the residual monomers and to sterilize the samples. Such design of post-processing conditions would allow standardization of sample preparation for cell studies. However, post-curing depends on the chemical composition (Alifui-Segbaya et al., 2017). Still, subsequent post-processing, in which the samples are washed in various solvents to remove the remaining uncured toxic monomers, is less dependent on chemical composition and can be generalized.

**FIGURE 1:**
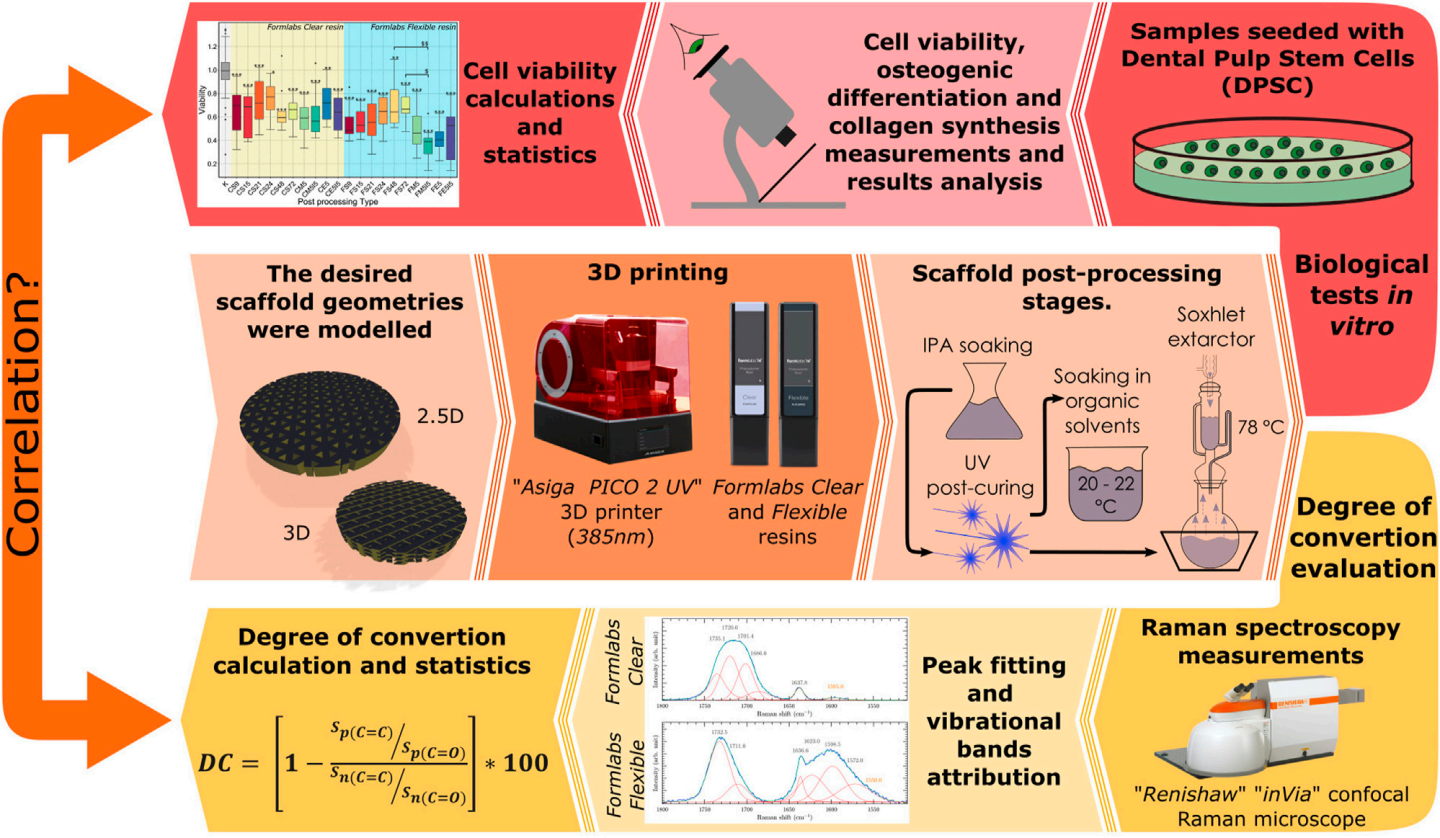
Graphical abstract of the entire study procedure representing framework geometries, material evaluated and polymerization device, post-processing methods and test types.

Nowadays, there are growing number of studies investigating different post-processing techniques to improve the biocompatibility of different samples. The values of DC are also being investigated in various ways, showing that this area is becoming increasingly important. However, the correlation between biocompatibility and the values of DC have not been studied in detail. Moreover, the different methods and materials used by different research groups do not allow a proper comparison. Therefore, our study aims to overcome this problem by using two widely used commercial resins (Figure 1) to compare the results obtained (in order to understand whether the post-processing trends are general or valid for specific materials). Two *Formlabs* resins were chosen - *Formlabs Clear* and *Flexible*. Although *Formlabs* also produces other resins for specific medical applications, such as *FormLabs Dental* and *BioMed*, these materials are intended for short-term use in contact with mucosal tissue and longer-term use with skin, not for cell growth or regenerative medicine. Although non-biomedical materials could, in principle, be less biocompatible, they could offer a greater contrast in biocompatibility variation that is influenced by post-processing.

We also studied for the first time how the Soxhlet extractor affects the biocompatibility of the samples (Figure 1). In addition, unlike all the previously mentioned references, we have looked more deeply into the suitability of the samples for tissue engineering, as we have investigated not only the viability of the cells but also their functionality and behaviour to understand if the scaffolds are biocompatible and suitable for cell adhesion, migration, proliferation and differentiation (Figure 1). Up to date, a post-treatment after photo-curing has not been studied and found to be associated with cell functionality in any other study. Finally, the correlation between cell viability and the values of DC was investigated and validated (Figure 1).

## 2 Materials and methods

### 2.1 Printing and post-processing of 3D scaffolds

**FIGURE 2:**
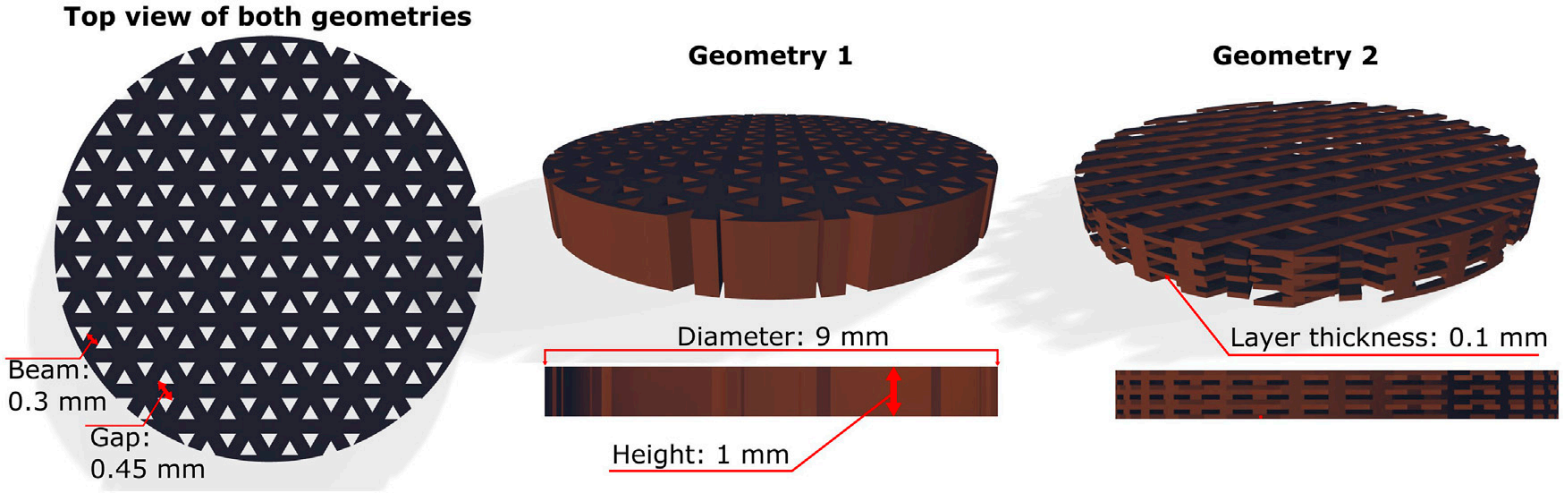
The geometries of the 3D scaffolds used in the study. The design of Geometry 1 can be described as 2.5D because the pattern does not change on the *z*-axis. The scaffolds of Geometry 2 represent different orientations of the superimposed printed beams in the *z*-axis.

The desired scaffold geometries were modelled using Autodesk Fusion 360 software and saved in. stl format. The scaffolds were modelled as disk-shaped structures with a diameter of 9 mm and a height of 1 mm. These dimensions were chosen to allow the specimens to fit into the well plates used for the biological studies. The internal geometry was a triangular-porous scaffold. The thickness of the beams was set at 0.3 mm with a gap of 0.45 mm (Geometry 1). For studies of differentiation and collagen synthesis, additional geometries (Geometry 2) were made (Figure 2). They had the same spacing and beam thickness, but different orientations of the beams were printed on top of each other on the *Z*-axis with a layer thickness of 0.1 mm.

Using the Asiga PICO2 39 UV 3D printer, the modelled structures were fabricated by DLP stereolithography using a LED light source with a wavelength of 385 nm. The scaffolds were fabricated out of two commercial *Formlabs* resins: *Formlabs Clear (FLGPCL02)* (hereafter referred to as *Clear*), and *Formlabs Flexible (FLFLGRO2)* (hereafter referred to as *Flexible*). *Formlabs Flexible (FLFLGRO2)* resin consisting of <0.9% diphenyl(2,4,6-trimethylbenzoyl)phosphine oxide (photoinitiator), 50%–70% urethane dimethacrylate (Formula: *C23H38N2O8*) and 30%–40% methacrylate monomers. This material has elastomeric properties and can be used for printing parts that can be easily bent and compressed. *Formlabs Clear* (FLGPCL02) resin consisting of <1% same photoinitiator, 25%–50% methacrylate monomers and 75%–90% methacrylate oligomers. Unlike *Flexible*, this material produces less flexible, stiffer parts, but its main advantage is its transparency, which makes it easier to use for biomedical research. Unfortunately, *Formlabs* has not disclosed either resin’s exact compositions of methacrylate monomers, oligomers and urethane dimethacrylate.

A systematic study was conducted to determine and optimize 3D parameters for printing with *Formlabs* resins on an Assiga printer. This study was based on the research by Skliutas et al. (2020) and further adapted for the specific case under examination. For the fabrication of Geometry 1 from these materials, several standard parameters were changed: slice height - 0.025 mm (both materials); waiting time (before exposure) of 2 s for *Flexible* resin and 2.5 s for *Clear* resin; exposure time of 0.41 s - *Flexible*, 0.5 s - *Clear*. For fabrication of Geometry 2: slice height - 0.01 mm (both materials); waiting time (before exposure) of 1.4 s for *Flexible* resin and 1.6 s for *Clear* resin; exposure time of 0.36 s - *Flexible*, 0.44 s - *Clear*.

After printing, all scaffolds were developed by soaking them in isopropanol for 30–40 min to wash out the monomers. They were then post-cured with UV light for 22 h (using the Asiga Flash Cure post-curing unit) to bond the uncured monomers. In the final phase, the scaffolds were post-cured in two ways. The first was soaking in isopropanol, methanol or ethanol. The other was soaking performed in a Soxhlet extractor. The Soxhlet extractor consists of three main parts: a condenser, a thimble, and a round bottom flask. The scaffolds were placed in the thimble. The thimble was then placed in the round bottom flask filled with ethanol. Finally, the flask was heated, causing the solvent to boil and evaporate. The vapour rose into the condenser to be cooled, condensed and dripped into the thimble. After 20 min, the thimble was full, and the ethanol flowed into the round bottom flask. The soaking protocols are explained in Table 1 with the abbreviations used later.

**TABLE 1:** Different post-processing methods of scaffolds and their abbreviations.

Soaking method	Abbreviation
Soaking samples using the Soxhlet extractor (solvent - ethanol) from 1 to 72 h	S1 - S72
Soaking in methanol and isopropanol for 1,2,3,4,5 days each	M1I1, M2I2, M3I3, M4I4, M5I5
Soaking in methanol for 4,5,6,7 days	M4 - M7
Soaking in ethanol for 5 days	E5
Soaking in ethanol and isopropanol for 5 days each	E5I5

### 2.2 Biocompatibility evaluation *in vitro*

Rat dental pulp-derived stem cells (DPSCs) were used for biocompatibility testing of the scaffolds. DPSCs were isolated and characterized as previously described (Alksne et al., 2019). Cell isolation procedures were approved by the Ethics Committee for Animal Experiments (Lithuania) No. G2-40, 2016-03-18. For experiments, DPSCs were maintained in Iscove’s Modified Dulbecco’s Medium (IMDM) supplemented with 10% fetal bovine serum (FBS) and 1% antibiotics (penicillin - 100 U/mL, streptomycin 100 mg/mL) at 37°C (all from Gibco, Paisley, United Kingdom). Cells were passaged twice weekly with 0.025% trypsin and 0.01% EDTA in phosphate-buffered saline (PBS) (Gibco, Paisley, United Kingdom). Before cell seeding, scaffolds were sterilized by washing them in 96% ethanol for 30 min using a shaker, then soaking in PBS for 2 × 1 hour, and finally drying under UV light (2 × 15 min, both sides). After sterilization, the scaffolds were incubated in IMDM supplemented with 10% fetal bovine serum and 1% antibiotics. After 22 h of incubation, the medium was collected for cytotoxicity assays. For this purpose, DPSCs were seeded at a density of 4.2 × 10^4^ cells/cm^2^ in 48-well plate 500 µL of cell suspension per well. After 24 h, the cell growth medium was replaced by the medium from the scaffolds. Cytotoxicity of the samples was assessed using the MTT (3-(4,5-dimethylthiazol-2-yl)- 2,5-diphenyltetrazolium bromide) (Sigma, St. Louis, MO, United States of America) assay, as described previously (Grigaleviciute et al., 2020). After 24 h of culture, the medium was discarded, and the samples were treated with MTT (1 mg/mL) and incubated at 37°C for 1 h. The MTT solution was then carefully replaced with 200 µL DMSO to dissolve the formazan. Subsequently, 100 µL of the formazan-DMSO solution was used to measure absorbance. The optical density at 545 nm was measured using a Varioskan Flash microplate reader (Thermo Scientific, Vantaa, Finland). The results were calculated as the ratio of cells grown in the test medium to cells grown in the control medium. At the same time, the biocompatibility of the scaffolds was evaluated by seeding DPSC at a density of 4.2 × 10^4^ cells/cm^2^ onto the scaffolds in 48-well plate 500 µL of cell suspension per well. The surface of a polystyrene tissue culture plate without scaffolds was used as a control surface. After 24 h of incubation, the MTT assay was performed as described above. Results were calculated as the ratio of cells grown on the tested scaffolds to the surface area of the polystyrene tissue culture plate.

In addition, a qualitative biocompatibility evaluation was performed using a scanning electron microscope (SEM) HITACHI TM -1,000 and a fluorescence microscope Olympus IX71. For SEM evaluation, cells were fixed by replacing the medium with a solution of glutaraldehyde (2.5%), formaldehyde (2%), and tannic acid (0.5%) and stored at room temperature for 30 min. The cells were washed three times with Dulbecco’s PBS and three times with distilled water. Then, the cells were dehydrated by adding ethanol solution at increasing concentrations (25, 50, 75, 90, and 100%), and then the critical point drying procedure (CPD) was performed. Finally, the samples with the fixed cells were coated with a 20 nm thick silver layer and subjected to SEM analysis. For cell viability analysis using a fluorescence microscope, 4 μg/mL of the dye mixture acridine orange/ethidium bromide (AO/EB) was added to the medium and incubated for 3 min. Finally, cell samples were washed with PBS and subjected to analysis.

### 2.3 Osteogenic differentiation

DPSCs were seeded at a density of 3.2 × 10^4^ cells/cm^2^ in 48-well plate 500 µL of cell suspension per well. To evaluate induced osteogenic differentiation on the tested surfaces. After 12 h, the growth medium was changed to an osteogenic medium consisting of DMEM medium supplemented with 10% FBS and 1% antibiotic mixture (penicillin (100 U/mL) and streptomycin (100 mg/mL)), 5 × 10^−8^ M dexamethasone, 25 μg/mL ascorbic acid, and 10^–2^ M β -glycerophosphate. One-half of the osteogenic medium was changed every second/third day. DPSCs were grown on scaffolds in a growth medium for spontaneous differentiation, with half of the medium changed every second/third day.

Alizarin red S staining (ARS) was performed to assess calcium-rich deposits in the cell cultures. After 14 days of induced and spontaneous differentiation, the medium was removed. Cells were then rinsed twice with PBS and fixed with 4% paraformaldehyde at room temperature for 15 min with gentle shaking (25 rpm). The residual fixative was removed, and the cells were washed with PBS. 2% ARS solution in deionized water (pH = 4.1-4.3) was added and incubated at room temperature for 20 min with gentle shaking (25 rpm). Subsequently, the ARS solution was discarded, and the samples were rinsed three times with deionized water and centrifuged at 100 g for 30 s (centrifuge HERMLE Labortechnik GmbH). Samples were analyzed microscopically to evaluate differentiation qualitatively (Olympus IX51). The monolayer appeared red/brownish when stained. For quantitative evaluation, ARS was dissolved in 5% perchloric acid; after 10 min, absorbance was measured at 490 nm using a Varioskan Flash microplate spectrophotometer (Thermo Scientific).

### 2.4 Collagen synthesis

To evaluate the intensity of collagen synthesis, cells were seeded at a density of 7.4 × 10^4^ cells/cm^2^ on the scaffolds. After 14 days, the medium was removed, and the samples were washed three times with cold PBS (500 μL) and then fixed with 4% formaldehyde (400 μL) for 15 min at room temperature. Cells were then stained with a solution of Sirius Red (1 mg/mL), incubated on a shaker (250 rpm) for 1 h, and then washed with a solution of HCl (400 μL). Finally, the dye was dissolved in a 0.1 M solution of NaOH (400 μL). To determine the amount of collagen, absorbance was measured at 550 nm using a Varioskan Flash microplate spectrophotometer (Thermo Scientific).

### 2.5 Evaluation of the degree of polymerization

To investigate the dependence of biocompatibility on the degree of polymerization, Raman micro-spectroscopy measurements of 3D microporous scaffolds were performed.

The micro-Raman spectra of uncured resin and printed scaffolds additionally cured and post-processed with UV light were recorded. Measurements were made using a “Renishaw” “InVia” micro-Raman spectrometer with a cooled (down to −70°C) multi-channel CCD detector. A diode laser with a wavelength of 785 nm and a grating of 1,200 lines/mm was chosen for excitation of the spectra. The laser beam was focused to a ∼1 μm spot using a “Leica N Plan” 50×/0.75 objective, with a power of 7.22 mW. Total accumulation time 400 s. The range of spectra obtained was 200–3200 cm^−1^ with a spectral resolution of ∼1 cm^−1^. The wavenumber was calibrated by the position of the Si band (520.7 cm^−1^).

The micro-Raman spectra were divided into smaller spectral regions, and background subtraction was performed using a cubic polynomial function. In addition, no smoothing procedures were applied to the data.

The fitting procedure was performed with mixed Lorentzian-Gaussian shape components using the software GRAMS/AI 8.0 (Thermo Electron Corp.) to obtain complete information about these spectral regions. Measurements of each different post-processed sample were repeated three times, and the DC was calculated and averaged.

The DC was calculated according to the following conventional equation (Žukauskas et al., 2015; Baldacchini et al., 2020):

$$DC=\left[1-\frac{S_{p\left(C=C\right)}/S_{p\left(C=O\right)}}{S_{n\left(C=C\right)}/S_{n\left(C=O\right)}}\right],$$ (1)

where S is the integrated area of the peaks, ‘p’–refers to the polymerized samples, and ‘n’–not polymerized. Here, calculations were performed by comparing the C=C stretching mode of methacrylate with a reference C=O band before and after photopolymerization.

### 2.6 Statistical analysis

All biological tests were repeated at least three times independently, with three samples within each experiment. Statistical analyses were performed using the R program package (RStudio version: 2022.07.2 + 576). Data are presented as median ± IQR or mean ± SD. Levene’s test was used to determine the homogeneity of variances (when the *p*-value was >0.05). If the *p*-value was not less than the significance level of 0.05, homogeneity of variances in the different treatment groups was assumed. In addition, to determine the normality of the data, the Shapiro-Wilk test was used (when the *p*-value was >0.05). Significant differences were determined using one-way analysis of variance (ANOVA) (viability test) and Kruskal–Wallis one-way analysis of variance (differentiation and collagen synthesis tests). Post hoc Tukey HSD (viability test) and Conover-Iman (differentiation and collagen synthesis tests) were used to assess statistically significant data differences for normality distributed data. Welch’s ANOVA was used to identify significant differences in the degree of conversion tests. Then, the Games-Howell *post hoc* test was employed to assess the statistical significance of the observed DC data differences. Statistically significant changes were considered where *p*-values were <0.05. The Ggplot2 library was used for graph preparation, and statistically significant differences in graphs are marked with * (or *$*) signs. * (or *$*) - *p* < 0.05, ** (or *$$*) - *p* < 0.01 and *** (or *$$$*) - *p* < 0.001.

## 3 Results

### 3.1 Printing and post-processing of 3D scaffolds

**FIGURE 3:**
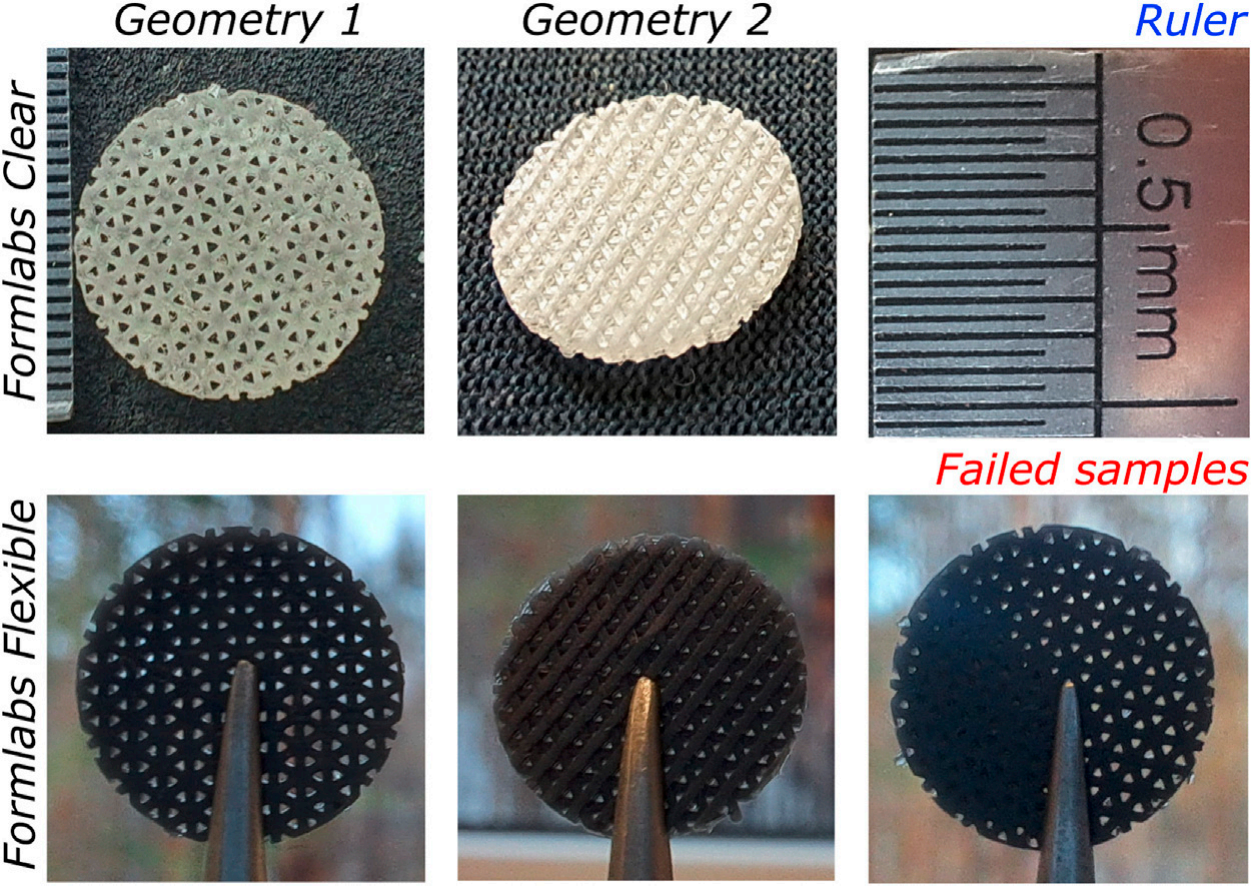
Images of 3D-printed scaffolds used for biocompatibility evaluation: Geometry 1 (left) and Geometry 2 (centre) made of *Clear* (top) and *Flexible* (bottom) resin. Also, an example of a failed sample (bottom right).

Two geometries of 3D scaffolds were printed from two resins: *Formlabs Clear* and *Flexible*. After printing the samples from the *Flexible* resin, it was observed that some of the pores of the scaffold were partially filled, as shown in Figure 3. This was not observed in the case of *Clear* material. Therefore, printing the *Clear* resin scaffolds was much easier than printing the *Flexible* scaffolds. After post-processing, it was found that the *Clear* resin scaffolds yellowed upon additional UV exposure. However, the geometry of the specimens remained noticeably unchanged after each post-processing step.

### 3.2 Biocompatibility of the scaffolds

**FIGURE 4:**
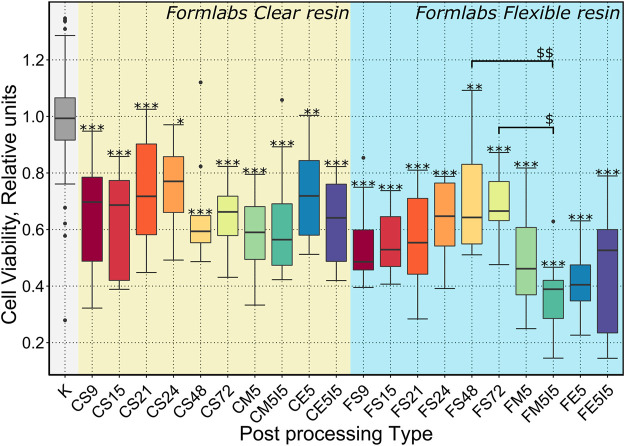
Results of a quantitative biocompatibility study. The cell viability results presented depending on the treatments performed after the additional UV exposure (22 h): S9, S15, S21, S24, S48, S72 - soaking in a Soxhlet extractor for 9, 15, 21, 24, 48, 72 h ; M5 - soaking in methanol for 5 days; E5 - soaking in ethanol for 5 days; M5I5 - soaking in methanol and isopropanol for 5 days; E5I5 - soaking in ethanol and isopropanol for 5 days F - *Formlabs Flexible*, C - *Formlabs Clear*. K - control. *** indicates a *p*-value of < 0.001 for the samples compared to the control, while *$$* indicates a *p*-value of < 0,01 between the indicated samples and * (*$*) indicates a *p*-value of < 0,05.

To evaluate the biocompatibility of scaffolds made from *Formlabs Clear* and *Flexible* resins depending on the type of post-processing, soaking in Soxhlet extractor for 6, 9, 12, 15, 18, 21, 24 h and soaking in methanol for 4, 5, 6, 7 days (Table 1) was chosen. The MTT assay for indirect determination of viable metabolically active cells showed that even the longest soaking time used for post-processing of the tested scaffold may not have been sufficient to elute the toxic monomers of both resins. Therefore, we decided to extend the soaking times in the Soxhlet extractor by another 48 and 72 h after UV fixation. To assess the effect of the chosen organic solvent on the biocompatibility of the scaffolds, additional post-processed samples were examined, i.e., they were soaked in methanol or ethanol for 5 days and, in addition, some of these samples were soaked in isopropanol for 5 days after UV fixation. The data presented in Figure 4 showed that the biocompatibility of scaffolds made of *Formlabs Clear* resin did not increase significantly depending on the type of post-processing. For the *Flexible* resin, however, soaking in a Soxhlet extractor significantly affected the biocompatibility of the scaffolds.

**FIGURE 5:**
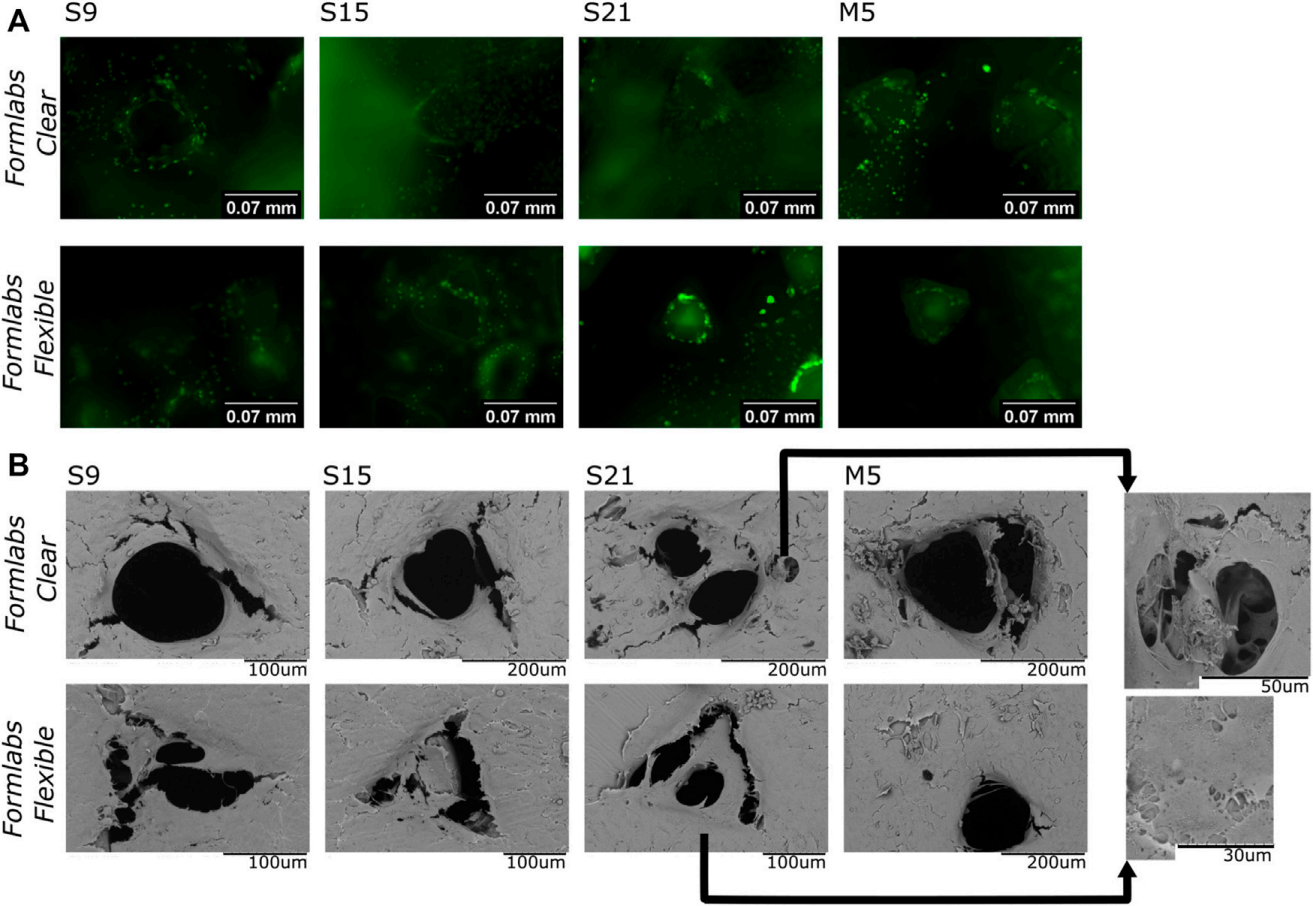
**(A)**-Results of fluorescence microscopy studies. The image shows a merged view by focusing the objective at the bottom of the well, the surface of the scaffold’s bottomand face, and the interior of the pores. **(B)**–SEM analysis (representative images) of cells grown on different samples. S9, S15, S21-soaking in Soxhlet extractor for 9, 15, and 21 h; M5-soaking inmethanol for 5 days.

Differential cell staining with AO/EB and fluorescence microscopy were used to assess cell distribution and migration in the scaffolds. The results showed that the cells were stained only green (cell death is indicated by orange staining (Kasibhatla et al., 2006), indicating that all cells were still viable after 72 h of growth on the scaffold (Figure 5A). These data confirmed the results of the MTT assessment. It was also observed that the cells in the pores of the scaffold tended to ‘climb’ up the walls. Thus, the results indicate that the scaffolds are suitable for cell adhesion and support cell migration.

The SEM analysis was performed to study the morphology and distribution of cells on the surface and their distribution in the pores of the scaffold. The images show that the cells adhere to the scaffold and form ‘bridges’ between the scaffold walls. In all tested samples, the surface was homogeneously covered with cells. Thus, the results confirm that the scaffold is a suitable base for cell attachment. Moreover, the “bridges” in the pores (Figure 5B) indicate that cells not only adhere to the surface, but can also migrate to the interior of the pores and attempt to adopt the physiologically preferred spatial orientation. Thus, the results confirm the biocompatibility of our samples.

### 3.3 Functionality of the scaffolds

**FIGURE 6:**
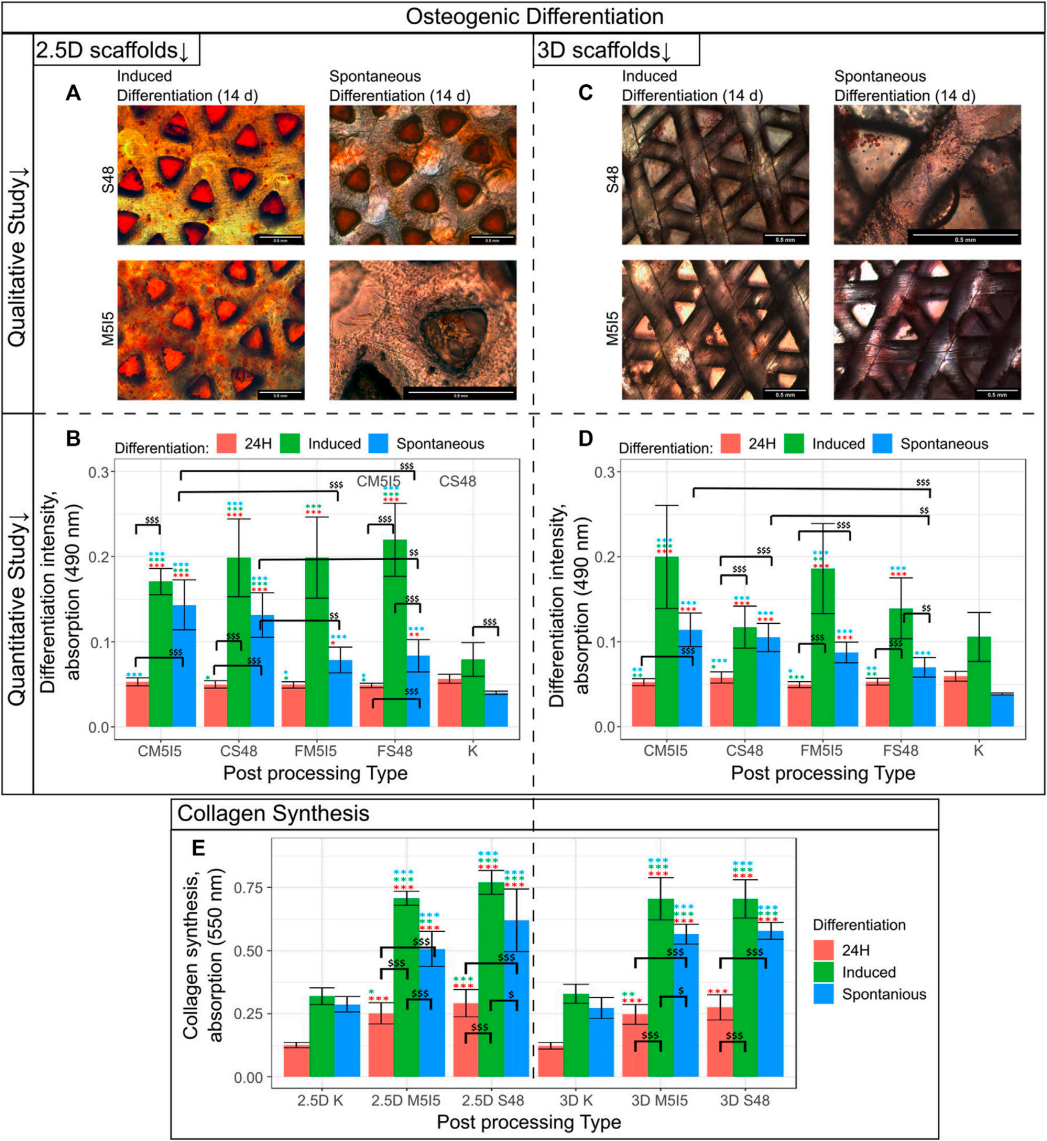
Results of the qualitative and quantitative study on osteogenic differentiation **(A)** Quantitative evaluation of cells grown on a 2.5D scaffold differentiation, where the intensity of differentiation corresponds to the absorbance at 570 nm; **(B)** Qualitative evaluation of cells grown on 2.5D *Clear* scaffold differentiation; **(C)** Quantitative evaluation of cells grown on a 3D scaffold differentiation, where the intensity of differentiation corresponds to the absorbance at 570 nm; **(D)** Qualitative evaluation of cells grown on a 3D *Clear* scaffold differentiation. S48 - soaking in Soxhlet extractor for 48 h, M5I5 - soaking in methanol and isopropanol for 5 days; C and F - *Clear* and *Flexible* materials. **(E)** Results of the collagen synthesis study.

The properties of the cell-supporting material significantly affect cell behaviour and function. Therefore, we investigated whether the properties of scaffolds made of *Formlabs Clear* and *Formlabs Flexible* resins are sufficient to induce osteogenic differentiation of DPSCs (hereafter - spontaneous differentiation). The results were compared with the intensity of osteogenic differentiation after treatment of DPSCs with specific osteogenic inducers. We compared the intensity of osteogenic differentiation on 2.5D and 3D scaffolds. The ARS staining showed that 2.5D and 3D scaffolds induced spontaneous differentiation regardless of the type of post-processing (Figures 6A,C). However, it is important to note that spontaneous differentiation was more intense on *Formlabs Clear* scaffolds. In contrast, the scaffolds from *Formlabs Flexible* tended to support induced differentiation better. However, the difference between *Formlabs Clear* and *Formlabs Flexible* was not statistically significant. Mineralization of the extracellular matrix (ECM) was also confirmed by microscopic analysis (Figures 6B,D).

Collagen synthesis and remodelling are essential for the maintenance of tissue homeostasis. Therefore, we examined the effects of scaffold post-processing and geometry on the ability of DPSCs to synthesize and deposit collagen, a major component of the ECM. The results show no statistically significant difference between the 2.5D and 3D geometry of the scaffolds (Figure 6E). Also, the type of post-processing of the scaffolds had no significant effect on the intensity of collagen synthesis. It is worth noting that in the case of induced differentiation, cells tend to deposit a higher amount of collagen. However, even in the case of spontaneous differentiation, the amount of collagen deposited was twice as high as in the control population.

### 3.4 Evaluation of the degree of polymerization

**FIGURE 7:**
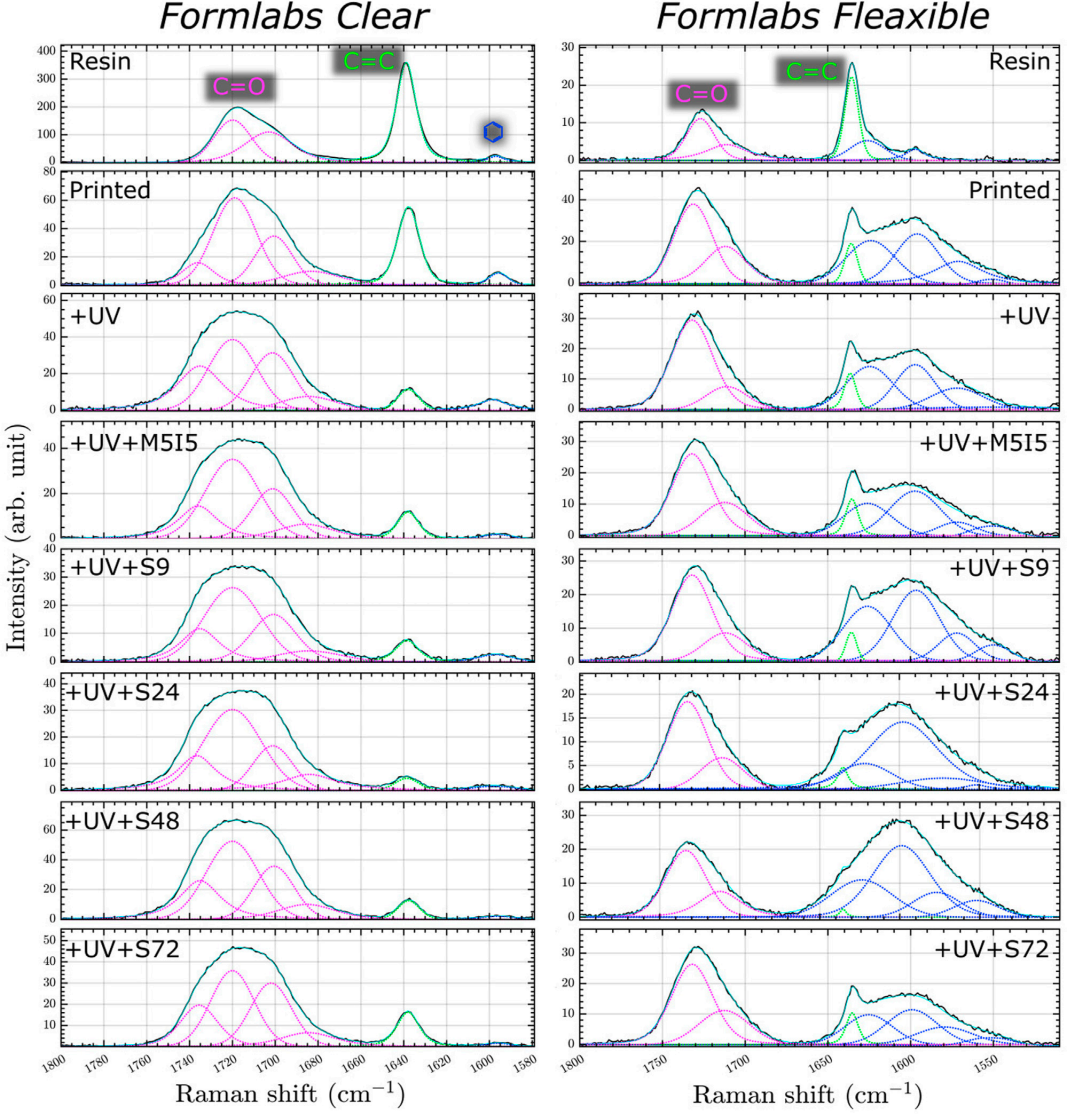
Micro Raman spectra of resin and 3D printed scaffolds: just printed, additionally UV post-cured (UV), post-cured and differently post-washed.

Two spectral regions between 1580–1800 cm^−1^ for *Clear* resin and 1510–1800 cm-1 for *Flexible* resin were selected for analysis of the micro-Raman spectra because these ranges contain a suitable value for the degree of polymerization. In the Raman spectra of *Clear* resin during processing, we observe three bands with changing properties (Figure 7). All these bands were approximated with a mixed Gaussian-Lorentz function. The most intens e band in the spectrum of the raw *Clear* resin is located at 1638.5 cm^−1^ and belongs to the C=C stretching vibrations of the methacrylate. The broad band at 1717 cm^−1^ is assigned to the stretching vibrations of a carbonyl group (C=O). The low-intensity band at 1596 cm^−1^ is assigned to the stretching vibrations of the aromatic C=C bond (Socrates, 2004) from the photoinitiator (Žukauskas et al., 2015). Similarly with the *Flexible* crude resin, here we observe bands at 1725, 1635.5, and 1596 cm^−1^ assigned to the valance of the carbonyl group, the C=C stretching vibration of the methacrylate, and the aromatic C=C of the initiator molecule, respectively.

In the micro-Raman spectra of *Clear* resin, the intensity of the C=C band decreases after polymerization. An even more pronounced decrease is observed when the printed samples are additionally post-treated with UV and soaked in organic solvents in two ways: with or without a Soxhlet extractor. This shows that during the photopolymerization reaction, the monomers combine to form a polymer structure - double C=C bonds turn into single C-C bonds (Žukauskas et al., 2015; Baldacchini et al., 2020). In addition, the double bonds are reduced when the monomers are washed out. The other change observed during polymerization is the shape and composition of the C=O band. The carbonyl band became wider and after polymerization, we have four components in the carbonyl vibrational band instead of two. Since the supplier did not provide complete information about the monomers and oligomers, we can only assume that four slightly different carbonyl bonds were formed during the polymerization process. The *Flexible* resin after the polymerization reaction showed the same tendency with the band at ∼1635.5 cm^−1^, while the carbonyl band with two components at ∼1732 and ∼1712 cm^−1^ remained the same. The band with the lower frequencies was assigned to the hydrogen-bonded C=O group, and the other to the “free\dprime carbonyl band (Dong and Ozaki, 1997).

As a result, the sum of the bands at ∼1717 cm^−1^ for the *Clear* resin was chosen as a reference for calculating the value of the degree of polymerization. Like the C=C band of the methacrylate group, the intensity of the aforementioned aromatic C=C band decreases and shifts to the blue side. The same trend of spectrum band change is observed in the samples of *Flexible* resin (Figure 7).

**FIGURE 8:**
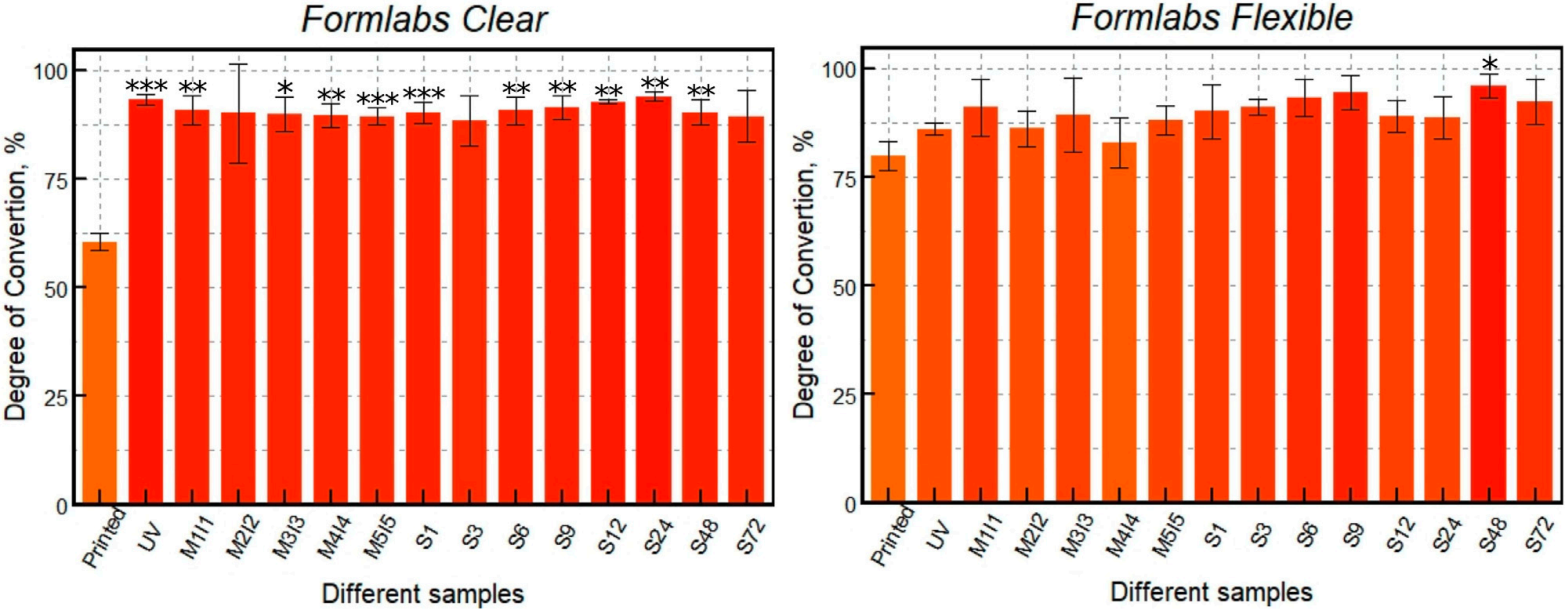
DC of 3D printed scaffolds: just printed, additionally UV post-cured (UV), post-cured and differently post-washed. *** indicates a *p*-value of < 0.001, ** - *p* < 0,01 and * - *p* < 0,05 for post-processed samples compared to the just printed ones.

Based on the dependencies shown in Figure 7, the degree of polymerization was calculated by comparing the areas of the slightly changing stretching oscillation bands of the carbonyl group (C=O) with the area of the C=C stretching oscillations of the methacrylate using Formula (1). The results show (Figure 8) no significant statistical difference between the differently post-processed samples. However, for the *Clear* resin, a significant statistical difference can be seen between the printed sample and almost all of the differently post-processed samples. For the scaffolds made of the *Flexible* resin, on the other hand, only one post-processed sample (S (48)) demonstrated a statistically significant difference compared to the sample immediately after printing.

## 4 Discussion

In this study, scaffolds were printed from two different resins, *Formlabs Clear* and *Formlabs Flexible*, to test the efficiency of different post-processing conditions. It is important to note that printing *Flexible* resin using the Asiga PICO2 39 UV 3D printer was much more challenging than printing *Clear* resin. The parameters of the scaffolds printed from *Flexible* resin were less reproducible, and the results had larger variations than the values for *Clear* resin.

The type of post-processing can affect cell fate when scaffolds are used for *in vitro* or *in vivo* studies, and also affects the physical-mechanical properties of the scaffold (Štaffová et al., 2022). A previous (Grigaleviciute et al., 2020) study reported that the most effective way for improving the biocompatibility of 2D-printed polymer samples is additional UV irradiation and prolonged soaking in polar solvents. Other studies (Kim et al., 2020; Bayarsaikhan et al., 2021) showed that cell viability and DC gradually increased with the duration of UV post-curing. However, further UV irradiation may result in no significant changes or even deterioration of biocompatibility and DC values. In addition, sample DC and biocompatibility values depend on the post-curing device used (Bayarsaikhan et al., 2022). Different results are obtained depending on the absorption spectra of the materials used. Therefore, different materials may require special post-curing equipment to achieve optimal results. To overcome this drawback, this study proposes to use the same device for post-curing different materials for a specific time.

Post-processing methods involving prolonged soaking in organic solvents (e.g., isopropanol, methanol) allow the unpolymerized monomers in the inner layers of the structure to be reached and washed out. Previous studies have shown a positive correlation between cell viability and scaffold washing time (Grigaleviciute et al., 2020; Hwangbo et al., 2021). In addition, washing of the specimens in an ultrasonic bath was shown to improve cell proliferation significantly (González et al., 2020). The use of other soaking methods may increase the biocompatibility and DC of scaffolds. We propose and validate the employed approach of soaking the samples in a Soxhlet extractor. The sample is immersed in a vessel with a solvent that is replaced with fresh solvent at each cycle (approximately 20 min, thus in our experiment from 3 to 216 cycles). This is a way more efficient option than simple soaking due to laminar flow of fresh solvent many times over. While some studies have used the Soxhlet apparatus for post-treatment of samples (Sirrine et al., 2018; Azman et al., 2020; Kaalberg et al., 2021), the effect of different post-processing times on biocompatibility and DC has not been investigated. Therefore, this study aimed to investigate whether the Soxhlet extractor could be more effective than other employed methods. The obtained results showed that the values for cell viability and DC improved up to a certain point with additional post-processing with the Soxhlet extractor. However, the values did reach saturation and did not improve further, may have even started to decrease slightly.

Next, this study examined the effects of scaffold post-processing on the biocompatibility of the resins’ and the functionality of the alive cells, including their ability to synthesize extracellular matrix and engage in cell differentiation. To our knowledge, no other study has investigated the relationship between post-treatment and cell functionality. Materials and scaffolds used for *in vitro* or *in vivo* applications must be biocompatible and affect cellular differentiation and migration in a cell type-specific manner (Murphy et al., 2013). Comparison of induced and spontaneous differentiation showed that scaffolds made of *Formlabs Clear* better supported differentiation of DPSCs toward the osteogenic lineage than *Formlabs Flexible*. These effects were not dependent on the type of post-processing. Furthermore, post-processing of the scaffolds also did not significantly affect the intensity of collagen synthesis. These data suggest that the selected post-processing methods can effectively wash out toxic unpolymerized monomers and support cell properties over time.

Micro-Raman analysis of the two resins *Clear* and *Flexible* was performed and the DC was calculated (Figure 8). The results show that different processing methods and resin choice led to variations in the degree of polymerization. First, the monomers in both resins were shown to be successfully converted to polymers with additional UV post-curing, resulting in average DC values of 60.5% for polymerized *Clear* resin and 80% *Flexible* resin. After additional UV post-curing, the DC value of *Clear* resin increased to 93%, while that of *Flexible* resin increased to 86%. It is important to note that the additional UV post-curing was more effective for the *Clear* resin than for *Flexible*. However, the DC values of *Clear* resin after UV fixation and additional soaking with and without Soxhlet extractor did not change significantly. When comparing different soaking methods, the DC values differed by only about 4.5%. Other trends in DC values can be observed for scaffolds made of *Flexible* resin. Soaking the samples in methanol and isopropanol for 5 days resulted in a DC value of 88%. However, when a Soxhlet extractor was used to soak the samples in ethanol, a higher degree of polymerization (96%) was obtained, indicating that this post-processing method is more effective in removing residual monomers after polymerization. Thus, the results of this study show that a high degree of polymerization is achieved during UV fixation of scaffolds printed from *Clear* material. Additional soaking methods do not increase it significantly. In contrast, the DC value of *Flexible* resin scaffolds after UV post-curing is lower than that of *Clear*. Although soaking *Flexible* resin scaffolds in methanol and isopropanol achieved a high DC, the highest DC (96%) was obtained with a Soxhlet extractor.

**FIGURE 9:**
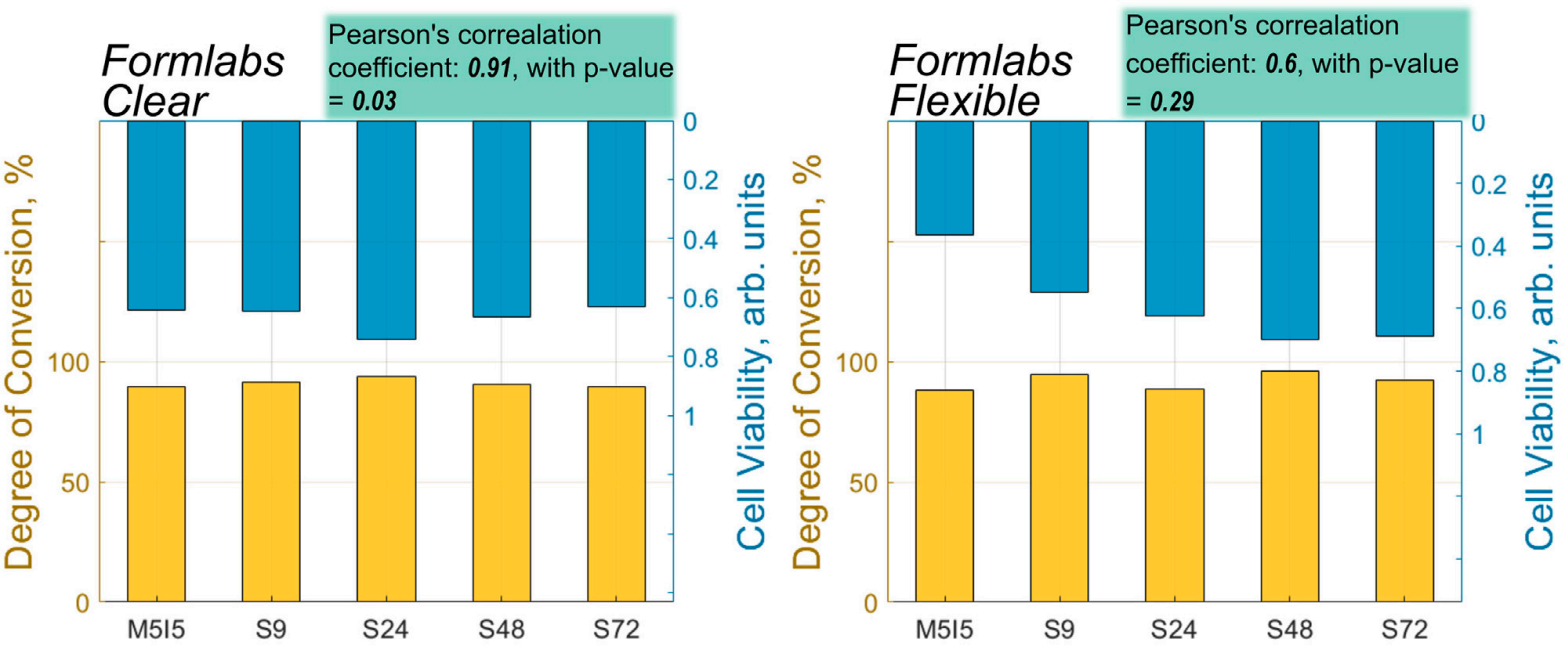
A correlation between the values of DC and cell viability of different post-processed samples made from *Flexible* and *Clear* resins.

The performed analysis of the correlation between the values of cell viability and DC showed that the correlation coefficient was high for both *Flexible* and *Clear* resins (*Clear* = 0.91, *Flexible* = 0.6) (Figure 9). The statistically significant value of the correlation, however, was observed only in the case of the scaffolds printed from the *Clear* resin, with a *p*-value of less than 0.05. As mentioned above, the *Flexible* resin was more difficult to process. The micro-Raman spectra of the *Flexible* resin showed significant variation across the scaffold volume, such that they did not polymerize equally well throughout their volume. The problematic processing of the resin could be the reason why the correlation results were not statistically significant. In addition, the DC values show that the post-processing combinations used were more effective for the *Clear* resin than for the *Flexible* resin. So, a well-controlled (easily and predictably polymerizable) resin shows correlation, while a difficult-to-control (more demanding and less repeatable) resin does not exhibit statistical significance in correlation. This could be because the print quality cannot be specified precisely, leading to different results for each print and making it difficult to determine accurate values for DC and biocompatibility.

In summary, this study introduces the use of the Soxhlet extractor as a new technique for post-processing samples that are further used for biomedical applications. Moreover, this research advances the field by investigating the dependence of biocompatibility on post-processing methods and its relationship with the degree of polymerization. Specifically, we have calculated and provided correlation values and conducted a comprehensive biocompatibility analysis. In addition, we have considered not only the viability of the cells but also their functionality and behaviour. Our further next goal is to gain a deeper understanding of the interplay of these factors that can guide the development of improved 3D-printed scaffolds for tissue engineering purposes.

## 5 Conclusion

A systematic study of the biocompatibility of optically 3D printed micro-porous scaffolds, their improvement by post-processing and analysis with Raman micro-spectroscopy revealed.

1. The biocompatibility of the photopolymerized 3D micro-porous materials depends on post-processing steps by minimizing the content of remaining non-crosslinked monomers. It can be increased with additional UV exposure followed by multiple rinses in the Soxhlet extractor.
2. The enhancement of biocompatibility was observed for both of the widely studied acrylate-based photopolymer materials and was more pronounced for *Flexible* (which was associated with less repeatable photo-structuring), but statistically more reliable for *Clear* (corresponding to stiffer printed structures).
3. The high correlation between biocompatibility and DC was found in the case of *Clear* resin (correlation coefficient: 0.91, with *p*-value = 0.03), but for scaffolds made from *Flexible* resin, the correlation coefficient was relatively high, 0.6, but statistically unreliable (*p*-value = 0.29), which could be due to the easier processing of more rigid structures than elastic ones.
4. Post-processing proved to be essential for supporting spontaneous cell differentiation and collagen synthesis. This suggests that selection of appropriate post-processing conditions may accelerate the use of synthetic polymers for *in vitro* and *in vivo* applications, including tissue engineering.

We foresee the Raman micro-spectroscopy as an efficient tool for optimizing the light exposure conditions and post-processing of samples required for the best *in vitro* result. Thus, it is a fast way to confirm the photo-curing effectiveness by the applied the exposure conditions. It allows the parameters of novel photopolymer materials for 3D printing to be examined and optimized even before scaffold geometries are investigated in terms of iterative and time-consuming statistical cell studies.

## Data Availability

The original contributions presented in the study are included in the article/Supplementary Material, further inquiries can be directed to the corresponding authors.
