# Supplementary material for: Biocompatibility enhancement via post-processing of microporous scaffolds made by optical 3D printer
Source: Front Bioeng Biotechnol. 2023 Apr 12;11:1167753. doi: 10.3389/fbioe.2023.1167753 (PMC10130666; doi:10.3389/fbioe.2023.1167753)
Supplement: Supplementary file 1 [file DataSheet1.PDF]

# Supplementary Material

SEM images taken from the bottom of the scaffold are shown in Figure S1.

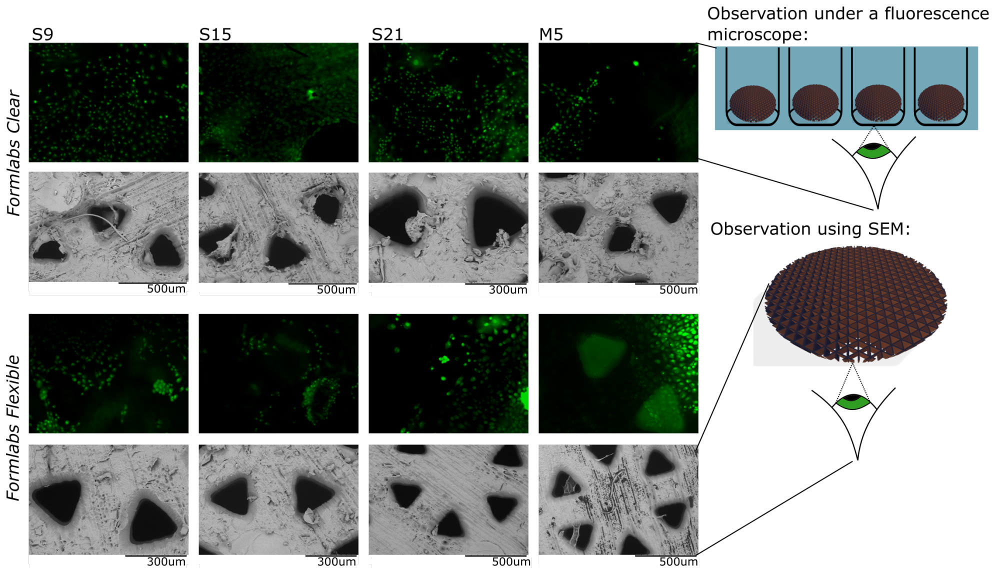


**Figure S1.** Fluorescence microscopy (colour images) and SEM (monochrome images) analysis of cells grown in different samples (representative images). SEM photos were taken photographing the sample from below, and fluorescence photos were taken by focusing the lens on the bottom of the well. S9, S15, S21 - soaking in a Soxhlet extractor for 9, 15, and 21 hours; M5 - soaking in methanol for five days.
